# Estimated Costs Associated With Radiation Therapy for Positive Surgical Margins During Radical Prostatectomy

**DOI:** 10.1001/jamanetworkopen.2020.1913

**Published:** 2020-03-31

**Authors:** Alberto Martini, Kathryn E. Marqueen, Ugo Giovanni Falagario, Nikhil Waingankar, Ethan Wajswol, Fahad Khan, Nicola Fossati, Alberto Briganti, Francesco Montorsi, Ashutosh K. Tewari, Richard Stock, Ardeshir R. Rastinehad

**Affiliations:** 1 Department of Urology, Icahn School of Medicine at Mount Sinai, New York, New York; 2 Department of Urology, Vita-Salute San Raffaele University, Milan, Italy; 3 Institute for Healthcare Delivery Science, Department of Population Health Science and Policy, Icahn School of Medicine at Mount Sinai, New York, New York; 4 Department of Pathology, Icahn School of Medicine at Mount Sinai, New York, New York; 5 Department of Radiation Oncology, Icahn School of Medicine at Mount Sinai, New York, New York

## Abstract

**Importance:**

Prostate cancer is the most common malignant neoplasm among men and is the one with the highest positive surgical margin (PSM) rate. This high rate is due to the difficulty in balancing the risk of extraprostatic disease and excising periprostatic structures, which ultimately affects patients’ quality of life. In the case of a PSM, the appropriateness of adjuvant radiation therapy (aRT) should be discussed. The financial burden of PSMs on health systems has not been investigated.

**Objective:**

To estimate the financial costs associated with a PSM during radical prostatectomy on the basis of the odds of undergoing aRT.

**Design, Setting, and Participants:**

This cohort study used data on men with prostate cancer from the US National Cancer Database (January 1, 2010, through December 31, 2015). Data were requested in March 2019, accessed in April 2019, and analyzed in August 2019.

**Exposure:**

Treatment with radical prostatectomy followed by aRT, if indicated.

**Main Outcomes and Measures:**

The attributable risk fraction of PSMs on undergoing aRT was estimated from a logistic regression with aRT administration as the outcome. The analysis was adjusted for patients’ socioeconomic and demographic characteristics and tumor characteristics. The aRT cost for the year 2019 was calculated using the Medicare Physician Fee Schedule and the Hospital Outpatient Prospective Payment System. The fraction of this cost attributable to a PSM was estimated according to its attributable risk fraction.

**Results:**

In total, 230 175 men were identified (median [interquartile range] age at diagnosis, 62.0 [56.0-67.0] years). Overall, 22.8% of the patients had a PSM. Patients with PSMs were more likely than those without PSMs to be older (median [interquartile range] age, 62.0 [56.0-66.0] years vs 62.0 [57.0-67.0] years) and nonwhite (9320 patients [17.8%] vs 29 872 patients [16.8%]), to have higher comorbidity scores (1604 patients [3.1%] vs 4884 patients [2.7%] with a Charlson-Deyo Comorbidity Index score ≥2) and worse tumor characteristics (category T3 and T4 disease, 26 394 patients [50.3%] vs 36 040 patients [20.3%]), and to have lower socioeconomic indicators (median annual income <$30 000, 5708 patients [10.9%] vs 17 874 patients [10.1%]; proportion of individuals without a high school degree in the area ≥29%, 6925 patients [13.2%] vs 22 648 patients [12.7%]). In addition, PSMs were documented more frequently at nonacademic institutions than academic ones (31 702 patients [60.5%] vs 20 714 patients [39.5%]). A total of 11 585 patients (5.0%) underwent aRT, and 7698 of them (3.3%) had a PSM at the final pathology examination. When controlling for patients’ socioeconomic and demographic characteristics and tumor characteristics, men with PSMs were more likely than those with negative margins to undergo aRT, with an odds ratio of 3.79 (95% CI, 3.63-3.96; *P* < .001). The attributable risk fraction of the presence of a PSM on aRT was 44% (95% CI, 42%-45%). The attributable cost of a PSM was calculated as $17 356 (95% CI, $16 567-$17 751). Assuming 60 000 prostatectomies in 2019 and similar trends of PSM and aRT, the overall health burden attributable to PSMs was calculated to be $52 068 000 (95% CI, $49 701 000-$53 253 000).

**Conclusions and Relevance:**

The estimated aRT cost attributable to the presence of a PSM was $17 356, resulting in $52 068 000 in spending on aRT in 2019. Strategies to reduce PSMs could be associated with a reduction in the overall health costs of surgically treated PCa.

## Key Points

**Question:**

What are the costs associated with positive surgical margins (PSMs) during radical prostatectomy?

**Findings:**

In this cohort study of 230 175 US men, the attributable cost of a PSM was $17 356. The overall health burden attributable to PSMs was estimated to be $52 068 000 each year.

**Meaning:**

The financial burden of PSMs is substantial, and efforts in reducing the rate of PSMs could be associated with a reduction in the overall health costs associated with surgically treated prostate cancer.

## Teaser

This cohort study estimates the financial costs associated with a positive surgical margin during radical prostatectomy according to the odds of undergoing adjuvant radiation therapy.

## Introduction

One of the goals of the surgical treatment for prostate cancer (PCa) along with oncological control is to provide patients with an optimal quality of life.^1^ In an effort to achieve that, surgeons must attempt a conservative dissection, allowing for maximal preservation of the structures surrounding the prostate, including the neurovascular bundles. The extent of the dissection will ultimately influence urinary continence and erectile function.

Considering the fact that surgeons walk on a fine line, trying to balance the risk of extraprostatic disease and the risk of resecting through tumorous tissue, the frequency of positive surgical margins (PSMs) is higher in men with prostate cancer compared with those with other malignant neoplasms.^2^ Also, compared with the most common malignant neoplasm among women (ie, breast cancer), the PSM rate for surgically treated PCa is almost 4 times higher.^2^ Because approximately 1 of 7 men will receive a diagnosis of PCa in their lifetime, the prognostic and financial implications of a PSM are obvious.^2^

The PSM rate ranges from 10% to 25% and has been slightly fluctuating over time.^3,4^ The introduction of robotic surgery initially led to an increase in the rate of PSMs, which later decreased in the postdissemination era. Recently, the use of preoperative multiparametric magnetic resonance imaging has been linked to a reduction in the PSM rate,^5^ but thus far no randomized study has validated this observation, to our knowledge.

The National Comprehensive Cancer Network and American Urological Association guidelines suggest discussing the administration of adjuvant radiation therapy (aRT) for patients with PSMs, because the presence of this single factor has the highest association with recurrence in this population.^6,7^ The European Association of Urology guidelines recommend the administration of aRT to individuals with a documented PSM in the case of locally advanced PCa.^8^ Nevertheless, aRT is still underused,^9^ and retrospective data suggest that surveillance followed by salvage radiation therapy in cases of recurrence may be a valid alternative.^10^ Although the association of a PSM with a patient’s prognosis is heterogeneous and depends on tumor category and grade,^11^ it is evident that this occurrence has a heavy burden on the health care system. In this study, we aimed to estimate the cost of a PSM by using the US National Cancer Database (NCDB) to calculate the attributable risk fraction (ARF) of a PSM on aRT while incorporating 2019 reimbursement data.

## Methods

### Patient Population

Data for this study were abstracted from the NCDB. Data were requested in March 2019, accessed in April 2019, and analyzed in August 2019. The version of the NCDB that was used corresponded to the version available in April 2019. The NCDB is a joint program of the Commission on Cancer of the American College of Surgeons and the American Cancer Society. The NCDB contains data originating from more than 1500 US hospitals and incorporates approximately 70% of all newly diagnosed cases of cancer. Details regarding the NCDB have been previously reported.^12^ We queried the PCa NCDB for patients who underwent radical prostatectomy and had complete data on clinical, socioeconomic, demographic, pathology, and treatment covariates.

These data encompass deidentified information. The institutional review board of Icahn School of Medicine at Mount Sinai approved their use for the purpose of the study and waived the need for informed consent. This study follows the Strengthening the Reporting of Observational Studies in Epidemiology (STROBE) reporting guideline.^13^

### Variables Definition

We considered patient socioeconomic characteristics, demographic factors, and tumor characteristics. Demographic variables included age, race, and Charlson-Deyo Comorbidity Index score. Socioeconomic factors included individual and neighborhood-level factors, including type of facility, primary payer, median annual income, proportion of individuals without a high school degree in the area, and distance from the facility.^14^ Tumor characteristics included year of diagnosis, tumor category, Gleason score, lymph node invasion, and surgical margins status. On pathology, neoplastic tissue in contact with the inked margin denoted a PSM. Adjuvant radiation therapy was defined as the beginning of radiotherapy within 6 months of surgery.

### Statistical Analysis

Descriptive statistics were generated; frequencies and proportions were reported for categorical variables, and medians and interquartile ranges were reported for continuous variables. Differences between medians and across frequencies were evaluated with the Kruskal-Wallis and the χ^2^ test, respectively.

Our statistical analysis *sensu stricto* consisted of 2 main steps. First, we estimated how much the presence of a PSM at final pathology was associated with the subsequent administration of aRT. Second, using the same regression model, we estimated the percentage and associated 95% CIs of how much of the administration of aRT can be attributed to the presence of a PSM.

For the first purpose, a multivariable logistic regression was fitted with aRT as the outcome. The role of PSMs in determining the administration of adjuvant treatments was investigated after adjusting for patient socioeconomic and demographic factors and tumor characteristics. Thereafter, the ARF of a PSM on the subsequent odds of undergoing aRT was calculated. This estimate (the ARF) reflects how much of the treatment (in this case aRT) can be attributed to a single variable, in our case the presence of a positive resection margin.^15^

Statistical analyses were performed using Stata statistical software version 14 (StataCorp). All tests were 2-sided with a significance level set to *P* < .05.

### Cost Estimation

In an effort to provide an estimate regarding the cost of the presence of a PSM, we considered the reimbursement rates for the year 2019 in the US in accordance with the Hospital Outpatient Prospective Payment System and the National Payment Amount for the Medicare Physician Fee Schedule. We considered the reimbursement for a conventional intensity-modulated radiation therapy as aRT for patients with PCa (ie, 70.2 Gy administered in 39 fractions). This was chosen because intensity-modulated radiation therapy is the most widely adopted treatment, while also compensating for the higher cost of proton treatment and lower cost of external beam radiation therapy.^16^ The total amount of the reimbursement was multiplied by the ARF of the presence of a PSM in determining the administration of aRT. This provided us an estimate of how much of the cost of aRT can be attributed to a PSM.

## Results

Overall, 230 175 men with complete data were identified. Patients received a diagnosis of PCa between January 1, 2010, and December 31, 2015. The median (interquartile range) age at diagnosis was 62.0 (56.0-67.0) years. At presentation, 186 793 patients (81.2%) did not have any comorbid conditions (Charlson-Deyo Comorbidity Index score, 0). On pathology, 167 741 patients (73.0%) presented with organ-confined disease (category T2), whereas 62 434 patients (27.2%) had non–organ-confined disease (category T3 or T4). Complete descriptive characteristics of the patient population are provided in Table 1. Overall, the PSM rate was 22.8%. The patients who underwent aRT were predominantly white (9434 white patients [81.4%] vs 1645 black patients [14.2%] and 506 patients [4.4%] of other race/ethnicity), had private insurance (6682 patients [57.7%] with private insurance vs 4903 patients [42.3%] with all other types of insurance), had high income (5299 patients [45.7%] with median annual income ≥$46 000 vs 1277 patients [11.0%] with median annual income <$30 000), and had high education levels (4704 patients [40.6%] with proportion of individuals without a high school degree in the area <14% vs 1584 patients [13.7%] with proportion of individuals without a high school degree in the area ≥29%).

**Table 1.** Descriptive Characteristics of the Patient Population

Characteristic	Participants, No. (%)			*P* value
	Overall (N = 230 175)	Adjuvant radiation therapy		
		No (n = 218 590)	Yes (n = 11 585)	
Age at diagnosis, median (interquartile range), y	62.0 (56.0-67.0)	62.0 (56.0-67.0)	62.0 (57.0-67.0)	<.001
Race				
White	190 983 (83.0)	181 549 (83.1)	9434 (81.4)	<.001
Black	29 741 (12.9)	28 096 (12.9)	1645 (14.2)	
Other	9451 (4.1)	8945 (4.1)	506 (4.4)	
Charlson-Deyo Comorbidity Index score				
0	186 793 (81.2)	177 416 (81.2)	9377 (80.9)	.10
1	36 894 (16.0)	35 051 (16.0)	1843 (15.9)	
2	5122 (2.2)	4842 (2.2)	280 (2.4)	
≥3	1366 (0.6)	1281 (0.6)	85 (0.7)	
Facility type				
Community cancer program	11 158 (4.8)	10 355 (4.7)	803 (6.9)	<.001
Comprehensive community cancer program	85 499 (37.1)	80 688 (36.9)	4811 (41.5)	
Academic or research program	101 020 (43.9)	96 618 (44.2)	4402 (38.0)	
Integrated network cancer program	32 498 (14.1)	30 929 (14.1)	1569 (13.5)	
Primary payer				
Not insured	3395 (1.5)	3141 (1.4)	254 (2.2)	<.001
Private insurance or managed care	142 581 (61.9)	135 899 (62.2)	6682 (57.7)	
Medicaid	5310 (2.3)	4880 (2.2)	430 (3.7)	
Medicare	74 894 (32.5)	70 952 (32.5)	3942 (34.0)	
Other government	3995 (1.7)	3718 (1.7)	277 (2.4)	
Annual median income quartiles in 2000, $US				
<30 000	23 582 (10.2)	22 305 (10.2)	1277 (11.0)	<.001
30 000-34 999	35 657 (15.5)	33 783 (15.5)	1874 (16.2)	
35 000-45 999	61 006 (26.5)	57 871 (26.5)	3135 (27.1)	
≥46 000	109 930 (47.8)	104 631 (47.9)	5299 (45.7)	
Individuals in the area with no high school degree in 2000, quartiles, %				
≥29	29 573 (12.8)	27 989 (12.8)	1584 (13.7)	<.001
20-28.9	46 527 (20.2)	44 020 (20.1)	2507 (21.6)	
14-19.9	54 133 (23.5)	51 343 (23.5)	2790 (24.1)	
<14	99 942 (43.4)	95 238 (43.6)	4704 (40.6)	
Distance from facility, median (interquartile range), miles^a^	14.5 (6.4-36.9)	14.8 (6.5-37.7)	10.9 (4.9-24.2)	<.001
Year of diagnosis, median (interquartile range)	2012 (2011-2014)	2012 (2011-2014)	2013 (2011-2014)	<.001
Tumor category				
T2	167 741 (73.0)	165 622 (75.0)	2119 (18.0)	<.001
T3	61 948 (27.0)	52 621 (24.0)	9327 (81.0)	
T4	486 (0.2)	347 (0.2)	139 (1.2)	
Gleason score				
≤6	57 588 (25.0)	57 173 (26.0)	415 (4.0)	<.001
7	145 946 (63.0)	139 853 (64.0)	6093 (53.0)	
8-10	26 641 (12.0)	21 564 (10.0)	5077 (44.0)	
Lymph node invasion				
Absent	223 051 (97.0)	213 335 (98.0)	9716 (84.0)	<.001
Present	7124 (3.0)	5255 (2.0)	1869 (16.0)	
Surgical margins status				
Negative	177 759 (77.0)	173 872 (80.0)	3887 (34.0)	<.001
Positive	52 416 (23.0)	44 718 (20.0)	7698 (66.0)	

^a^ To convert miles to kilometers, multiply by 1.61.

Table 2 displays the distribution of PSM frequencies over patients’ socioeconomic, demographic, and pathology factors. Patients with PSMs were more likely than those without PSMs to be older (median [interquartile range] age, 62.0 [56.0-66.0] years vs 62.0 [57.0-67.0] years) and nonwhite (9320 patients [17.8%] vs 29 872 patients [16.8%]), to have higher comorbidity scores (1604 patients [3.1%] vs 4884 patients [2.7%] with a Charlson-Deyo Comorbidity Index score ≥2) and worse tumor characteristics (category T3 and T4 disease, 26 394 patients [50.3%] vs 36 040 patients [20.3%]), and to have lower socioeconomic indicators (median annual income <$30 000, 5708 patients [10.9%] vs 17 874 patients [10.1%]; proportion of individuals without a high school degree in the area ≥29%, 6925 patients [13.2%] vs 22 648 patients [12.7%]). In addition, PSMs were documented more frequently at nonacademic institutions than academic ones (31 702 patients [60.5%] vs 20 714 patients [39.5%]). The Figure displays the frequency of PSMs according to facility type over the years.

**Table 2.** Frequencies of Surgical Margins According to Patients’ Socioeconomic Characteristics and Tumor Factors^a^

Characteristic	Positive surgical margin, patients, No. (%)		*P* value
	No (n = 177 759)	Yes (n = 52 416)	
Age at diagnosis, median (interquartile range), y	62.0 (56.0-66.0)	62.0 (57.0-67.0)	<.001
Race			
White	147 887 (83.2)	43 096 (82.2)	<.001
Black	22 585 (12.7)	7156 (13.7)	
Other	7287 (4.1)	2164 (4.1)	
Charlson-Deyo Comorbidity Index score			
0	144 994 (81.6)	41 799 (79.7)	<.001
1	27 881 (15.7)	9013 (17.2)	
2	3861 (2.2)	1261 (2.4)	
≥3	1023 (0.6)	343 (0.7)	
Facility type			
Community cancer program	8260 (4.6)	2898 (5.5)	<.001
Comprehensive community cancer program	65 052 (36.6)	20 447 (39.0)	
Academic or research program	80 306 (45.2)	20 714 (39.5)	
Integrated network cancer program	24 141 (13.6)	8357 (15.9)	
Primary payer			
Not insured	2509 (1.4)	886 (1.7)	<.001
Private insurance or managed care	111 611 (62.8)	30 970 (59.1)	
Medicaid	3875 (2.2)	1435 (2.7)	
Medicare	56 775 (31.9)	18 119 (34.6)	
Other government	2989 (1.7)	1006 (1.9)	
Annual median income quartiles in 2000, $US			
<30 000	17 874 (10.1)	5708 (10.9)	<.001
30 000-34 999	27 122 (15.3)	8535 (16.3)	
35 000-45 999	46 778 (26.3)	14 228 (27.1)	
≥46 000	85 985 (48.4)	23 945 (45.7)	
Individuals in the area with no high school degree in 2000, quartiles, %			
≥29	22 648 (12.7)	6925 (13.2)	<.001
20-28.9	35 597 (20.0)	10 930 (20.9)	
14-19.9	41 499 (23.3)	12 634 (24.1)	
<14	78 015 (43.9)	21 927 (41.8)	
Distance from facility, median (interquartile range), miles^b^	14.8 (6.6-37.8)	13.6 (6.0-33.5)	<.001
Year of diagnosis, median (interquartile range)	2012 (2011-2014)	2012 (2011-2014)	<.001
Tumor category			
T2	141 719 (79.7)	26 012 (50.0)	<.001
T3	35 924 (20.2)	26 024 (50.0)	
T4	116 (0.1)	370 (1.0)	
Gleason score			
≤6	50 813 (29.0)	6775 (13.0)	<.001
7	111 044 (62.0)	34 902 (67.0)	
8-10	15 902 (9.0)	10 739 (20.0)	
Lymph node invasion			
Absent	174 233 (98.0)	48 818 (93.0)	<.001
Present	3526 (2.0)	3598 (7.0)	

^a^ Percentages in each category do not always total 100% because of rounding.

^b^ To convert miles to kilometers, multiply by 1.61.

**Figure.**
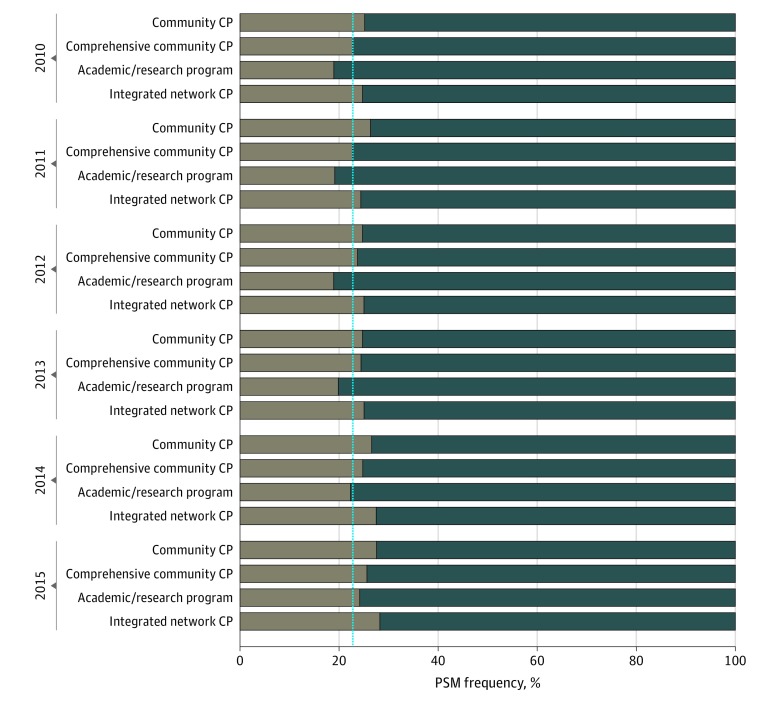
Frequency of Positive Surgical Margins (PSMs) According to Facility Type Over the Years Brown bars show the PSM rate, and blue bars show the negative surgical margin rate. The blue line displays the overall PSM rate in the study cohort (22.8%). CP indicates cancer program.

A total of 11 585 (5.0%) patients underwent aRT, and 7698 (3.3%) of them had a PSM on their final pathology examination. Of the 63 429 patients (28.0%) with locally advanced disease (T3 or N1), 26 654 patients (42.0%) had a PSM on their specimen and 9610 patients (15.0%) underwent aRT. Of the 166 746 patients (72.0%) with organ-confined disease, 25 762 patients (15.0%) had a PSM and 1975 patients (1.2%) underwent aRT. Of the men with a PSM, 26 012 had organ-confined category T2 disease (Table 2), and 2119 of them (or 1 of 13) underwent aRT (Table 1).

Overall, when controlling for patients’ socioeconomic and demographic characteristics and tumor factors, men with PSM had increased odds of undergoing aRT (odds ratio [OR], 3.79; 95% CI, 3.63-3.96; *P* < .001), compared with those with negative margins (Table 3). Other factors associated with undergoing aRT were tumor category T3 (OR, 2.64; 95% CI, 2.38-2.93) and T4 (OR, 7.01; 95% CI, 6.29-7.82), compared with category T2, and Gleason scores of 7 (OR, 6.36; 95% CI, 6.04-6.71) and 8 to 10 (OR, 6.54; 95 CI, 5.25-8.14) compared with scores of 6 or less. The ARF of the presence of a PSM on the administration of aRT was 44% (95% CI, 42%-45%).

**Table 3.** Multivariable Logistic Regression Predicting the Administration of Adjuvant Radiation Therapy

Covariate	OR (95% CI)	*P* value
Age at diagnosis, y	0.98 (0.98-0.98)	<.001
Race		
White	1 [Reference]	
Black	1.11 (1.04-1.18)	.001
Other	1.11 (1.01-1.23)	.04
Charlson-Deyo Comorbidity Index score		
0	1 [Reference]	
1	0.87 (0.82-0.92)	<.001
2	0.90 (0.78-1.03)	.11
≥3	0.96 (0.76-1.23)	.78
Facility type		
Community cancer program	1 [Reference]	
Comprehensive community cancer program	0.82 (0.75-0.90)	<.001
Academic or research program	0.57 (0.52-0.62)	<.001
Integrated network cancer program	0.63 (0.57-0.69)	<.001
Primary payer		
Not insured	1 [Reference]	
Private insurance or managed care	0.76 (0.65-0.88)	<.001
Medicaid	1.02 (0.85-1.22)	.84
Medicare	0.76 (0.65-0.88)	<.001
Other government	1.09 (0.89-1.33)	.40
Annual median income quartiles in 2000, $US		
<30 000	1 [Reference]	
30 000-34 999	1.00 (0.92-1.09)	.96
35 000-45 999	1.01 (0.93-1.10)	.80
≥46 000	1.06 (0.97-1.16)	.21
Individuals in the area with no high school degree in 2000, quartiles, %		
≥29	1 [Reference]	
20-28.9	1.07 (0.99-1.15)	.11
14-19.9	0.99 (0.91-1.08)	.81
<14	0.96 (0.88-1.04)	.32
Distance from facility, miles	1.00 (1.00-1.00)	<.001
Year of diagnosis	0.98 (0.97-1.00)	.01
Tumor category		
T2	1 [Reference]	
T3	2.64 (2.38-2.93)	<.001
T4	7.01 (6.29-7.82)	<.001
Gleason score		
≤6	1 [Reference]	
7	6.36 (6.04-6.71)	<.001
8-10	6.54 (5.25-8.14)	<.001
Lymph node invasion		
Absent	1 [Reference]	
Present	1.83 (1.72-1.96)	<.001
Surgical margins status		
Negative	1 [Reference]	
Positive	3.79 (3.63-3.96)	<.001

Abbreviation: OR, odds ratio.

We estimated that the reimbursement for intensity-modulated radiation therapy treatment was $39 446 for the year 2019. Thus, the attributable cost of a PSM estimated on the odds of receiving aRT was $17 356 (95% CI, $16 567-$17 751). This was obtained by multiplying the ARF and its associated 95% CI (0.44; 95% CI, 0.42-0.45) by the cost of intensity-modulated radiation therapy ($39 446).

In accordance with the national trend of prostatectomies per year from the national inpatient sample, we estimated that approximately 60 000 radical prostatectomies were performed in 2019. If 5% of the patients underwent aRT, the overall health burden attributable to PSMs would be $52 068 000 (95% CI, $49 701 000-$53 253 000). Assuming a similar distribution of organ-confined and locally advanced disease, approximately $9 372 240 ($8 946 180-$9 585 540) could be attributed to PSMs in organ-confined disease. This was obtained by multiplying overall health burden attributable to PSMs ($52 068 000 and associated 95% CI) by the rate of aRT in case of organ-confined disease (18%).

## Discussion

Because of the advent of robotic surgery, the rate of PSMs has been slightly decreasing over time; however, the overall PSM rate in the NCDB is still nonnegligible, with approximately 1 of 4 or 5 men who has a PSM after radical prostatectomy.^17^ Such PSMs translate into worse prognosis^11,18,19^ in terms of recurrence that is ultimately reflected in enormous health costs.

In this study, we have tried to estimate how much of the cost of aRT can be ascribed to the presence of PSMs on final pathology. To do so, we tried to predict the administration of aRT as the outcome of our multivariable analysis. Then, using the same model, we went backward in the prediction process and used the ARF of a variable (ie, PSM), to abstract how much of the outcome can be attributed to the same variable. After we obtained the ARF and associated 95% CIs, we multiplied this percentage by the cost of aRT to provide an estimate of the cost of a PSM.

In an effort to provide an estimate of how much of the cost of aRT can be attributed to PSMs, our primary analysis focused on the entire patient population who underwent radical prostatectomy between 2010 and 2015. Our model demonstrates that after adjusting for potential confounders, the presence of a PSM accounts for 44% of the decision to administer aRT following surgery. Of the cost of aRT, $17 356 could be attributed to the presence of a PSM on final pathology.

American Urological Association and National Comprehensive Cancer Network guidelines suggest discussing the appropriateness of the administration of aRT in patients with a PSM.^3,4^ However, the European Association of Urology guidelines suggest doing so in the case of non–organ-confined disease,^5^ and the present data seems to suggest that this is the preference among US urologists as well. In fact, it appears that approximately 1 of 13 men with a PSM in the context of organ-confined disease underwent aRT. Theoretically, these costs could be reduced with the implementation of well-structured training. In fact, although a PSM in the context of a non–organ-confined disease is sometimes inevitable, a PSM in organ-confined disease is the result of a breach in the prostatic pseudocapsule in the area where the tumor is located and is more representative of a surgeon’s mistake. One can argue that patients with non–organ-confined disease should undergo radiation therapy up front, and this would decrease the cost originating from a multimodal approach. However, patients with category T2 disease could potentially be cured with surgery, and the avoidance of PSMs would remove the costs associated with aRT and all other potential subsequent therapies for this group. Our secondary analysis, focused on patients with organ-confined disease, showed that $9 372 240 could be ascribed to intracapsular margins.

A recent study^20^ demonstrated that the risk of PSMs is dramatically reduced in both organ-confined and locally advanced disease after a learning curve of approximately 250 robotic procedures. Specifically, Bravi et al^20^ showed that the percentage of reduction in PSMs was greater in cases of non–organ-confined disease (approximately 25%), vs organ-confined disease (approximately 10%). A more structured training with the implementation of a specific curriculum could allow for potential PSM reduction and subsequent health care cost reduction.^21^ Although it is true that the introduction of robotic surgery has allowed for the reduction of the overall rate of PSMs, our findings suggest that this occurrence is still quite frequent even in the postdissemination era.

The rate of PSMs is sometimes considered as a proxy of the quality of the surgery performed and, ultimately, of a surgical center.^22^ We found that PSMs were more frequent in nonacademic centers and in older, nonwhite patients with more comorbidities and lower socioeconomic indicators, such as primary payer, median income, proportion of individuals without high school degree in the area, and distance from the facility. When taking all these factors into account, PSMs might also be a sign of health disparities that may warrant further research.

Our study, in keeping with previous findings, suggests that aRT is still underused. It is true that not all PSMs are equal and not all of them have the same prognostic outcome. Several studies have tried to address this point, aiming to define margins’ features that have greater implications in terms of recurrence. Thus far, the Gleason score in the margin location^23,24^ and a PSM length greater than 3 mm have been associated with a greater risk of biochemical recurrence,^18,21^ whereas multiple margins or a single margin greater than 3 mm have been associated with a greater risk of metastasis in cases of non–organ-confined disease.^11^

However, these data originate from analyses of retrospective studies and, to our knowledge, no prospective series has yet confirmed these results. The lack of available data describing margin features precluded us from taking this factor into account in our analysis.

### Limitations

This study has some limitations. Despite revealing a tremendous financial burden for health care systems, this analysis represents an underestimation of the true cost of PSMs in the real-world setting. In fact, PSMs are well known to be associated with biochemical recurrence.^18,21,25^ In addition, in the case of adverse pathology, PSMs are associated with a higher risk of developing metastasis during long-term follow-up.^26^ Our estimates do not capture the subset of patients who have been offered surveillance and subsequently received salvage treatments (either salvage radiation therapy and/or salvage androgen deprivation therapy) because those data are not collected in the NCDB. These factors render our estimate an underestimation of the true costs. In addition, the fact that the NCDB only captures approximately 70% of the newly diagnosed cases of PCa every year might influence our estimates.^12^

## Conclusions

To our knowledge, this study is the first to provide an estimate of the cost of a PSM. We estimated the aRT cost attributable to the presence of a PSM to be $17 356, resulting in $52 068 000 in spending on aRT in 2019. Even though these numbers represent an underestimation of the real PSM burden, they point to the fact that strategies to reduce PSMs could be associated with a reduction in the overall health costs of surgically treated PCa.
